# Intent to purchase IoT home security devices: Fear vs privacy

**DOI:** 10.1371/journal.pone.0257601

**Published:** 2021-09-21

**Authors:** Joey F. George, Rui Chen, Lingyao Yuan

**Affiliations:** ISBA Department, Ivy College of Business, Iowa State University, Ames, IA, United States of America; Southern Illinois University, UNITED STATES

## Abstract

The Internet of Things (IoT) is a widely hyped concept, with its focus on the connection of smart devices to the Internet rather than on people. IoT for consumers is often called the smart home market, and a large part of that market consists of home security devices. Consumers are often motivated to purchase smart home security devices to prevent burglaries, which they fear may lead to damage to their property or threats to their families. However, they also understand that IoT home security devices may be a threat to the privacy of their personal information. To determine the relative roles of fear and privacy concerns in the decision to purchase IoT home security devices, we conducted a survey of American consumers. We used the Theory of Reasoned Action as the theoretical basis for the study. We found that fear positively affected consumer attitudes toward purchasing smart home security devices, while concerns about privacy negatively affected attitudes. We found that attitudes toward purchase, the opinions of important others, and experience with burglaries all affected intent to purchase. We also found that the relationship between privacy concerns and intent to purchase is completely mediated by attitudes, while fear has both direct and indirect effects on intent.

## Introduction

The Internet of Things (IoT), with its focus on connecting devices instead of people via the Internet, is one of the most hyped information systems topics of the last decade [1]. IoT for consumers is often referred to as the ‘smart home’ market and includes such things as smart televisions, smart speakers (e.g. Alexa), thermostats, lighting controls, video doorbells, and security cameras. The global market for the smart home industry is projected to be worth over $150 billion USD by 2023, while the US market, with 45 million smart devices in 2019, will be worth $42 billion [2,3]. While most consumers (84%) prize the smart home’s convenience, 37% purchased IoT devices for home and family security. The global market for smart home security devices alone was $2.14 billion USD in 2018, and it is expected to increase to an estimated $5.05 billion by 2025 [4]. Certain smart security devices are popular–the most common home security device is a camera, and smart doorbells rank third [3]. Unit sales for cameras and smart doorbells increased by 125% between February 2017 and February 2018, and it is estimated that Ring, the Amazon owned maker of video doorbells and related security devices, sold more that 400,000 units in December 2019 alone [5,6].

Smart home security marketing mentions many reasons why a consumer would want to invest in such devices, but the marketing almost always appeals to fear. For example, safewise [7], an independent review site, answers the question “Do I Really Need a Security System?” with:

According to Alarm System Report, over 2 million home burglaries are reported in the United States each year and, on average, a burglary of a home in the U.S. occurs every 13 seconds-or about four burglaries a minute, 240 an hour, and nearly 6,000 a day.

Reolink, a vendor of home security cameras, lists in a blog 10 reasons why consumers need a home security system [8]. The first reason is “to secure your family and property.” The post then cites the same statistics about burglaries that appear on the safewise site quoted above. They go on to say that “FBI burglary rates of homes state that 1 in 3 homes without a security system will fall victim to a burglary as compared to 1 in 250 homes that do have a security system,” followed by videos–caught on Reolink cameras–of burglars attempting to break into a home and a car.

Yet consumers recognize there is a potential tradeoff between security and privacy. In a recent survey of over 1000 US consumers, 50.2% were most concerned about protecting their homes, but an almost equal proportion, 49.8%, were most concerned about protecting their personal data [3]. For example, Ring doorbells have become a privacy concern, with documented cases of smart doorbells leaking WiFi login credentials, with their known vulnerability to hackers, and the company’s practice of sharing users’ videos with law enforcement [9]. Perhaps the best-known case of a privacy breach from the use of a Ring product occurred in Mississippi in December 2019. An unknown man gained access to a Ring camera and watched an eight-year-old girl, asking her at one point to be his best friend, claiming he was Santa Claus [10].

To investigate the respective roles of fear of burglary and concerns over data privacy in the decision to purchase smart home security devices, we conducted a survey of potential consumers of such devices. We used the Theory of Reasoned Action [11] to form the theoretical framework for our study. We found that beliefs about fear affected the attitude toward purchasing the most, but beliefs about privacy also played a role. Attitude toward purchase and the views of important others affected purchasing intent about equally. Whether or not someone had personal experience with burglary also positively affected the intention to purchase smart home security devices.

In the next section, we review the literature and present the theoretical foundation for our study. We then describe our research design, data collection, and analysis. We end with a discussion of our findings and their implications.

## Theory and literature

The Theory of Reasoned Action (TRA) is a well-known and widely used theory for explaining individual intent to engage in a particular behavior [11,12]. Attitude toward the target behavior and subjective norms about engaging in the behavior are thought to influence intent. According to TRA, an individual’s performance of a certain behavior is determined by his or her intent to perform that behavior. Intent is informed by attitudes and subjective norms. According to Ajzen [13], attitudes are informed by beliefs about the behavior, and norms are informed by normative beliefs and motivation to comply. Specifically, attitude toward a behavior is shaped by behavioral beliefs, readily accessible in the memory of a decision maker, which refer to the subjective evaluations of those consequences following performing the behavior. Subjective norms are under the influence of normative beliefs that concern the perceived expectations of referent individuals or groups, for which the decision maker is motivated to comply. As a general theory, TRA does not specify the particular beliefs that are associated with any particular behavior, nor does it specify referent individuals or groups. Both of these tasks are left to the researcher and are dependent on the nature of the behavior in question.

**Fig 1:**
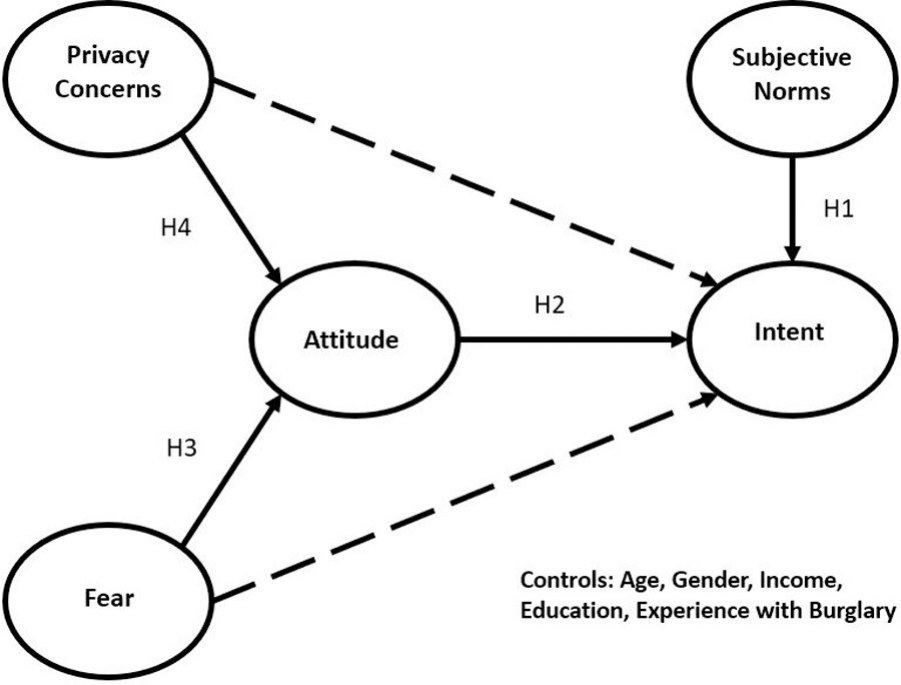
TRA model of intent to purchase IoT home security devices.

TRA has a long history of use in the social sciences. For example, a 1999 meta-analysis of 67 studies on the intent to use condoms found that attitudes toward condom use and subjective norms demonstrated medium to strong effect sizes [14]. TRA has been particularly useful as a framework for explaining the intent to purchase consumer goods. Recent examples include the intent to buy a Volkswagen [15]; environmentally friendly (green) products [16]; “pioneer” products (those produced by a first mover firm in a market) [17]; “functional” yogurt (a functional food is one to which nutritional value has been added) [18]; “upcycled” products (upcycled products create value out of old or discarded materials) [19]; foreign goods [20]; and participation in online fashion renting [21]. Because it is functional, relatively simple, and reliable, we use TRA as the framework for our investigation into the relative influence of fear and privacy concerns on the intent to purchase IoT home security devices (Fig 1).

Across a wide range of recent papers about the intent to purchase IoT devices, subjective norms and attitude were key factors in explaining intent [22–26]. Where subjective norms were investigated, the important others identified varied with context, as would be expected. Important others listed in these studies included retailers in general, competitors, and the media [22]; the generic “people important to me” [23]; and the generic “social circle” [25]. Where beliefs about purchasing were identified, both security [25,27–29] and privacy concerns [24–26,28] were key factors. In general, security concerns positively affected intent, while privacy concerns negatively affected it. Where included, price value either positively affected intent [25] or it had no effect [29].

Given these findings, and given the practical considerations for focusing on security and privacy concerns (mentioned earlier), we included both in the model as beliefs informing the attitude toward purchasing. Given all the different ways security concerns were operationalized, we decided to use fear as a surrogate. We did not include price value, given its general absence in other studies (in these IoT purchasing papers we reviewed, price value was not included in [22–24,26,27,30], the contradictory outcomes where it was included (no effect on intent in [29], but a positive effect on intent in [25]), and our inability to find or develop a suitable measure for the cost of smart home security devices. To cast a wide net for influential others, we decided to use friends, neighbors, family, and co-workers as important others. For control variables, we included age, gender, income, education, and experience with burglary.

Since its development in the 1970s, in many different studies across many different behaviors, the strong relationships between subjective norms and intent, and between attitude and intent, have become standard and expected. We therefore predict that:

***H1***: *Subjective norms about purchasing a home security IoT device positively influence the intent to purchase such a device*.
***H2***: *Positive attitudes towards the intent to purchase a home security IoT device positively influence the intent to purchase such a device*

Fear is an important construct in the literatures on how people respond to threats and on security, going back at least to its implicit inclusion in Protection Motivation Theory [31]. Rogers referred to fear as a “relational construct, aroused in response to a situation that is judged as dangerous and toward which protective action is taken” ([31], p. 96). In the case of deciding to purchase IoT home security devices, the situation that is judged as dangerous is having one’s home burglarized, potentially damaging one’s property and/or harming one’s family. The protective action is purchasing and installing home security equipment. In the information systems security literature, fear has become an explicit construct [32]. The fear appeal literature suggests that fear is responsible for attitude change [31], so we would expect those with the most fear to be the most attracted to IoT home security devices.

***H3***: *Fears over home security will positively affect attitudes toward purchasing an IoT home security device*.

Our other key belief informing attitude toward purchasing, privacy concerns, has been important in studies of consumer purchasing intention and behavior for decades [33–35]. IoT industry and government agencies such as the US FTC have all stressed the privacy issue when guiding consumers about their purchasing decisions [36]. A systematic review of the behavioral literature on privacy and e-commerce [37] found eight different themes. Three of these themes are directly related to IoT devices and privacy: 1) e-commerce benefits and consumer privacy, 2) privacy-related risks and concerns, and 3) information control and privacy. Related to the first theme is the idea that consumers are likely to give up some degree of privacy if they believe they are receiving a fair exchange. In this case, the exchange would be between security and privacy. For the second concern, perceived risk is driven by the likelihood of negative consequences. Fortes and Rita [38] found that privacy concerns were related to increased perceptions of risk, which affected attitudes toward engaging in e-commerce. Regarding the third concern, control over private information has been prominent in the privacy literature. Many times control is considered to be a contextual matter rather than a personal disposition. Given a focus on fair exchanges, risk and control (or lack of it), consumers with privacy concerns related to information technology tend to be less likely to purchase technologies that have the potential to provide private information to unwanted others. Unlike traditionally “dumb” devices, IoT devices are equipped with the ability to collect personal data such as personal identities, geolocations, habits, and physical conditions. When consumers are aware of the potential risks to the data collected by their IoT devices, they may be less attracted to such devices.

***H4*:***Beliefs about the perceived privacy risks of IoT home security devices will negatively affect attitudes toward purchasing them*.

A central principle of TRA is that the influence of beliefs on intent are mediated by attitudes toward the behavior in question. Direct paths from the beliefs to intent are typically not tested. We have added those direct paths in Fig 1 (dotted lines). After we test the TRA structural model, we will test it a second time with the direct paths included, to determine the extent to which beliefs about purchasing smart home security devices are mediated by attitudes towards purchasing such devices in their effect on intent to purchase. We did not suggest a hypothesis regarding the direct paths, as we have no basis from the literature for doing so.

## Research method & results

### Measurement

To test our models (Fig 1), we searched for an existing survey instrument that measured all of the relevant constructs. As none existed, we created our own instrument, beginning with a search for pre-existing measurement scales for as many constructs as possible. TRA does not specify specific behaviors or referent groups, so standard TRA measures must be modified for the behavior and referent groups under study. Here we needed to find and modify suitable measures for key aspects of TRA, including intent, attitude, and subjective norms. Measures for attitude and subjective norms were taken from [33], which was based largely on [39], and modified for the behavior in question, adopting Internet-of-Things home security devices. The scale for fear came from [40] and was modified for fear of having one’s home burglarized. Privacy risk measures were derived from [41] and modified to capture beliefs about the risks to privacy from adopting IoT home security devices. Measures for intent were developed by the authors, as we were unable to find any suitable scales that measured the intent to adopt IoT devices for home security. All items, except those for ‘attitude,’ were measured using a 7-point Likert scale, from ‘1’ for ‘strongly disagree’ to ‘7’ for ‘strongly agree.’ The four ‘attitude’ items used semantic differential seven-point scales, ranging from bad to good, foolish to wise, dislike to like, and unpleasant to pleasant. We used the question of “Has your home or that of someone you know been burglarized?” to capture the burglary experience of consumers to be surveyed. This measure was developed by the authors. It was measured on a five-point scale: 1 = in the past year; 2 = in the past two years; 3 = in the past three to five years; 4 = more than five years ago; 5 = never burglarized. Finally, we used standard measures for the other control variables: age, gender, education and income.

During the development of the survey instrument, the authors went through several rounds of revision. The original versions of the instrument contained multiple measures of some constructs. All of the items to be used in the instrument were subjected to a sort procedure conducted individually by three doctoral students and one professor. Each item was printed on a separate piece of paper, and the judges were asked to sort them into however many piles they thought were appropriate. They were then asked to name each pile. There was considerable overlap among the judges, but there was no complete consensus. Some judges had more piles of items than others, and the distribution of items across piles differed by judge. The areas where they did not agree pointed to potential problems. The authors revised the instrument, based on the feedback from the sorting procedure, until the instrument was stable. The authors made several distinct changes in the instrument: 1) They changed the measure for intent, dropping two items and adding three new ones; 2) They added income and education to the demographic variables; and 3) They added the item about whether or not the respondent’s home had been burglarized.

The revised instrument was then validated, beginning with a pilot study with 100 participants. The participants were recruited by Cint, a firm that provides access to over 40 million consumers worldwide, for a cost. To qualify for the survey, potential respondents had to indicate that they were not current users of IoT home security devices. The data collected during the pilot were subjected to tests of reliability and confirmatory factor analysis according to each construct and its antecedents. The authors decided to include all items in the pilot study in the primary data collection effort. In December 2017, data was collected through Cint from 229 U.S. consumers [42], using the same screening procedure used in the pilot. Demographics are shown in Table 1, and descriptive statistics for the measures are listed in Table 2. Based on an analysis of normal Q-Q plots for each variable, the data are normally distributed. Note that the sample skews heavily female. Both popular press and academic articles indicate that women are more likely than men to purchase IoT devices for safety (19% to 13%), while men are more likely to purchase IoT devices for energy savings [43,44]. Cannizzaro and colleagues [30] reported from their survey that while 57% of men owned smart home devices, 64% of women did. Given our interest here in privacy concerns in this study, it is interesting to note that the same authors reported that trust in privacy related to smart home devices was lower for women than for men.

**Table 1:** Sample demographics.

Gender	Education	Income
Female: 77.3%	Grad degree: 14.4%	<$50k: 55.5%
Male: 22.7%	College degree: 29.7%	$50k-$100k: 28.8%
	Some college: 27.5%	>$100k: 16.7%

**Table 2:** Descriptive statistics (N = 229 for all variables).

	Minimum	Maximum	Mean	Standard Deviation
Intent	1.00	7.00	3.28	1.66
Fear	1.00	7.00	3.52	1.73
Attitude	1.00	7.00	5.11	1.37
Privacy Concerns	1.00	7.00	4.61	1.15
Subjective Norms	1.00	7.00	3.52	1.39

### Measurement model

**Fig 2:**
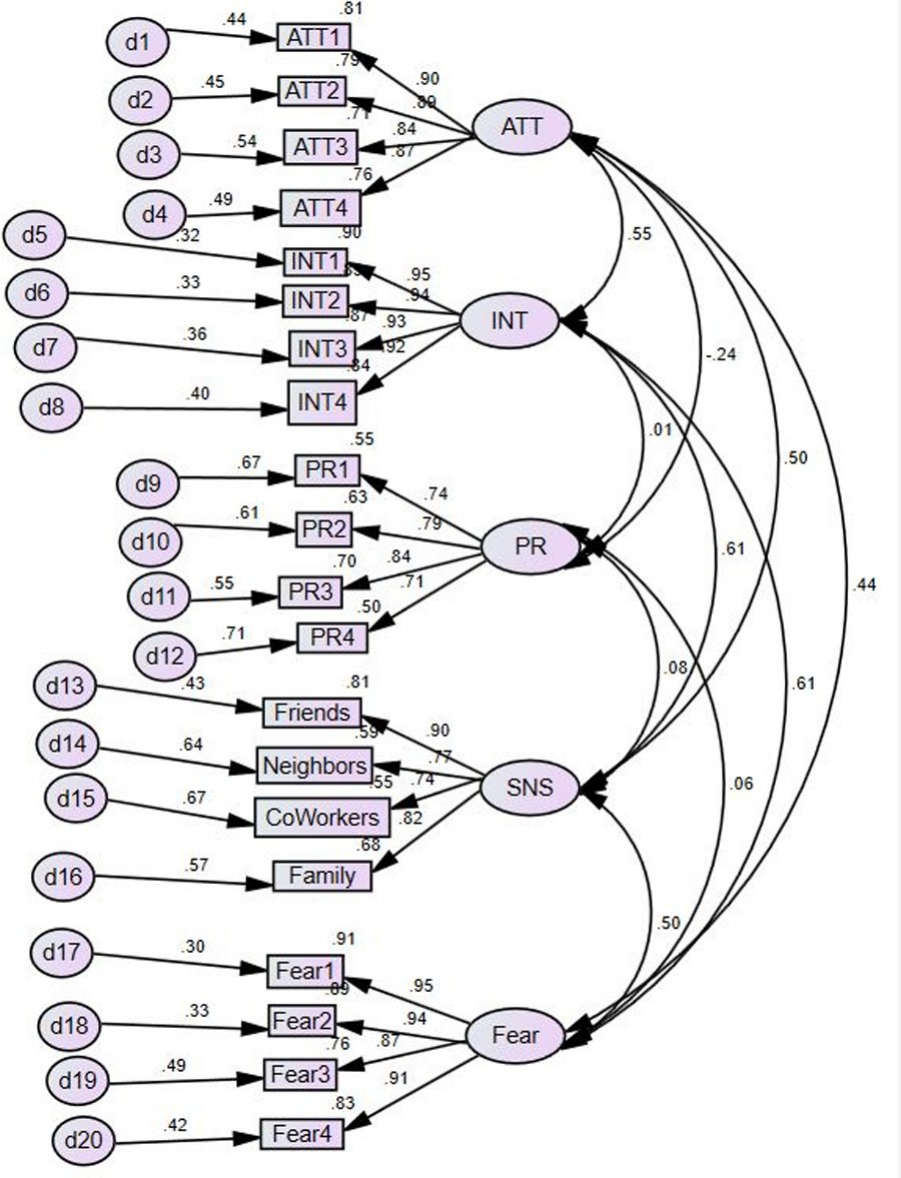
Confirmatory factor analysis results.

The measurement and structural models were both tested with AMOS 25, part of IBM’s SPSS statistical package. We ran a confirmatory factor analysis with the five constructs in our model (Fig 2). The data fit the model well (Table 3). The reliability statistics for the scales were all at acceptable levels, and the average variance explained (AVE) values were all above 0.5 (Table 4). The square roots of the AVEs for each construct exceeded the largest correlation for that construct (Table 5). In summary, the measurement model demonstrated good convergent and discriminant validity.

**Table 3:** Measurement model fit statistics.

Criterion	Fit statistic
Chi-square	231.109
DF	160
Chi-square/DF	1.444
p	.000
CFI	.983
NFI	.946
RMSEA	.044

**Table 4:** Measurement model convergent validity statistics (standardized regression weights).

Construct & item	λ	Cronbach’s α	CR	AVE
**Intention**		.965	.966	.875
INT1	.948			
INT2	.942			
INT3	.932			
INT4	.919			
**Attitude**		.929	.930	.770
ATT1	.900			
ATT2	.892			
ATT3	.845			
ATT4	.872			
**Privacy Concerns**		.853	.854	.596
PR1	.744			
PR2	.793			
PR3	.836			
PR4	.707			
**Fear**		.956	.957	.846
Fear1	.953			
Fear2	.943			
Fear3	.872			
Fear4	.909			
**Subjective Norms**		.883	.884	.657
Friends	.901			
Neighbors	.767			
Co-workers	.741			
Family	.823			

**Table 5:** Measurement model discriminant validity statistics.

	Intention	Attitude	Privacy	Fear	Subjective Norms
Intention	**.935**				
Attitude	.552	**.877**			
Privacy	.011	-.239	**.772**		
Fear	.611	.438	.062	**.920**	
Subjective Norms	.610	.499	.076	.496	**.810**

Following [45], we took procedural remedies to control for common method variance (CMV). Specifically, we (1) kept the survey anonymous to reduce respondents’ evaluation apprehension and to reduce the odds of respondents providing socially desirable, lenient, or acquiescent answers; (2) randomized the question order to control for priming effects and other biases related to question context or item embeddedness; and (3) as noted, improved scale items through the rigorous scale development process. With the survey data collected, we performed empirical tests to assess the extent of bias that may have resulted by CMV [46]. Following Lindell and Whitney [47], we partialed out a marker variable (a theoretically unrelated construct with three items: “I prefer blue to other colors,” “I like blue color,” and “I like blue clothes”) in our model and examined the correlations between this marker and all latent variables. Common method bias exists if the correlation between any of the latent variables and the marker is greater than 0.3, which is not the case in this study, as the highest correlation we observed was 0.109. Following Podsakoff et al. (2003), we compared the R^2^ changes of all endogenous variables in our model, before and after partialing out the above marker variable. Common method bias exists when there are significant changes of R^2^ values; we found the highest R^2^ value change in our data set to be 0.004. Common method bias is, therefore, not a threat to our study.

In addition, we tested for the possible presence of multicollinearity. Scatter plots showed that the data were normally distributed. Variance inflation factors for privacy concerns, fear, and subjective norms were all between 1.0 and 1.15, indicating multicollinearity was not a problem.

### Test of TRA structural model

**Fig 3:**
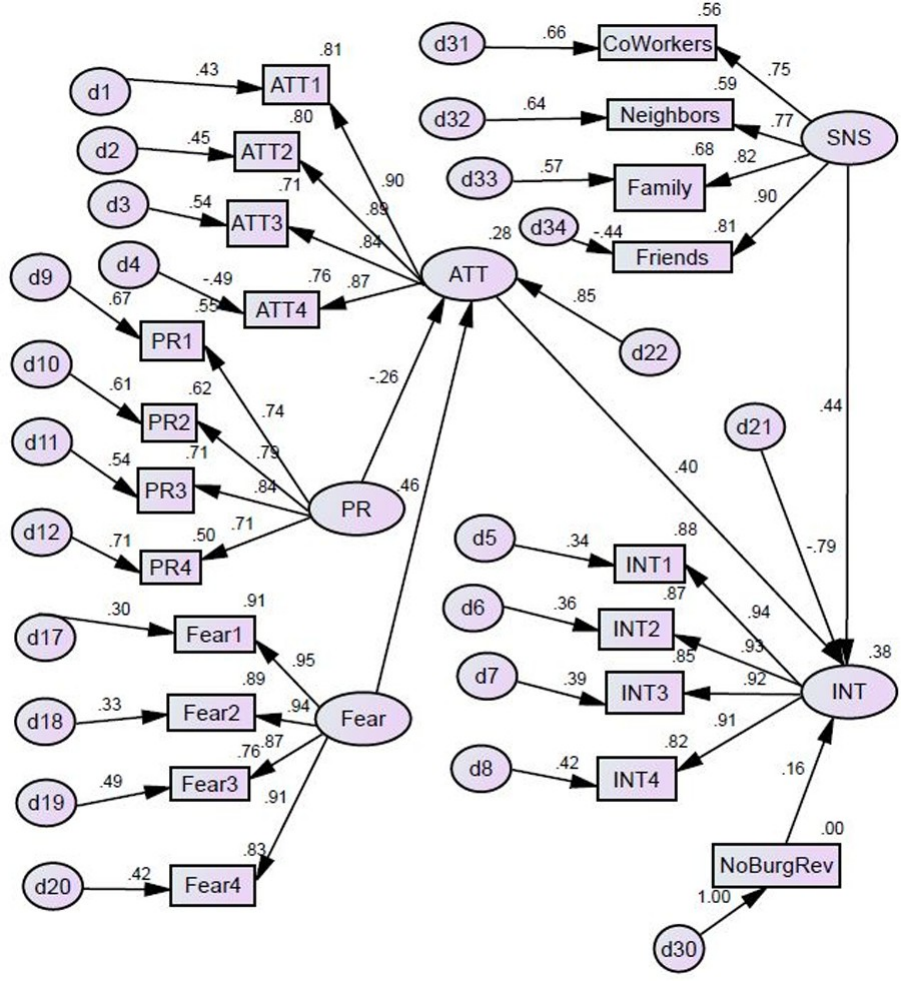
Evaluated TRA structural model.

The test of the structural model yielded a χ2 (185) = 386.8 (ρ < 0.000). Other indicators also pointed to a good fit of the data to the model: CFI = 0.951; NFI = 0.911; RMSEA = 0.069. Privacy concerns showed a negative and significant relationship with attitude toward purchasing IoT home security devices (β = - 0.26, ρ = 0.000). Fear showed a positive and significant relationship with attitude (β = 0.46, ρ = 0.000). Attitude (β = 0.40, ρ = 0.000) and subjective norms (β = 0.44, ρ = 0.000) both showed a positive and significant relationship with intent to purchase. We tested the relationships between each control variable and the measure of intent, and none of these relationships was statistically significant except experience with burglary (β = 0.16, ρ = 0.005), such that those with personal experience with burglary had more intent to purchase. The model explained 38% of the variance in intent to purchase an IoT home security device and 28% of the variance in attitude (adjusted R^2^). All four hypotheses were supported. The evaluated TRA model is shown in Fig 3.

### Test of mediation structural model

**Fig 4:**
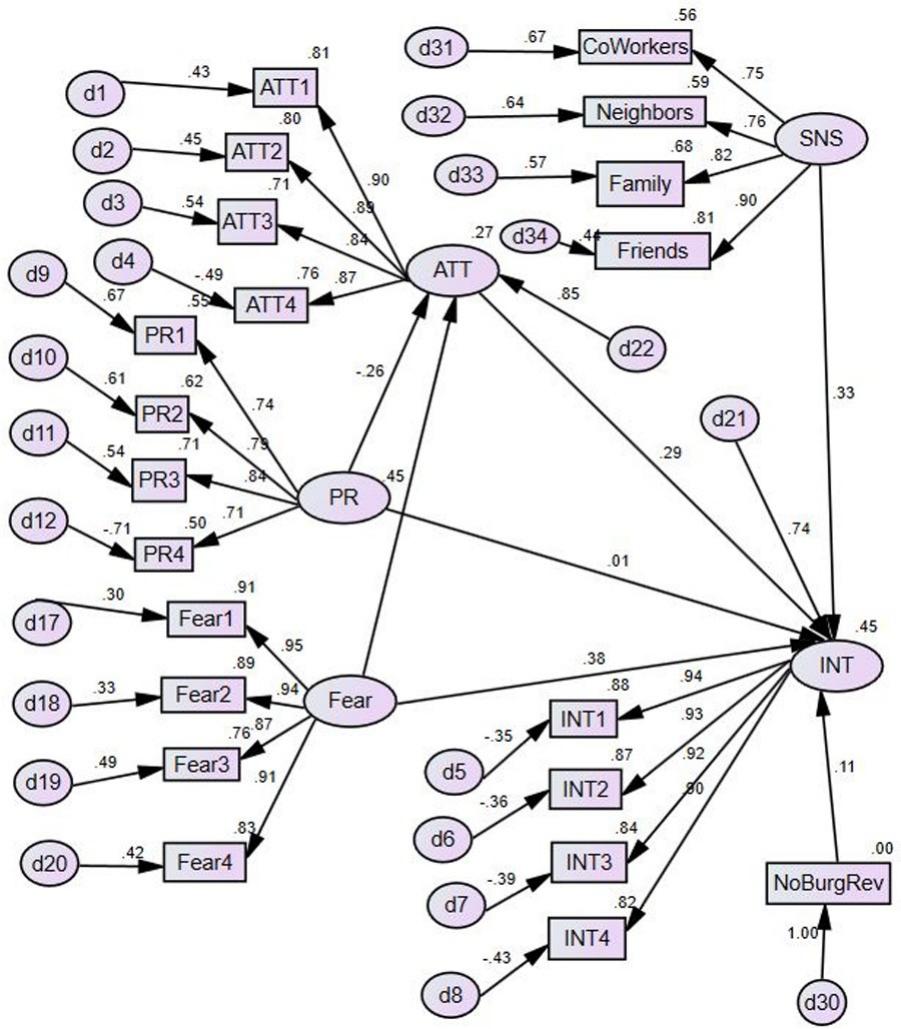
Evaluated mediated structural model.

Including the direct paths from privacy concerns and fear to intent to purchase allow us to test the extent to which attitudes mediate the effect of beliefs on intent (Fig 4). The test of the mediation structural model yielded a χ2 (183) = 354.4 (p < 0.000). The model was a good fit to the data (CFI = .959; NFI = .919; RMSEA = .064). Once again, attitude (β = 0.29, p = 0.000) and subjective norms (β = 0.33, p = 0.000) both demonstrated positive and statistically significant relationships with intent. The effect on attitude of privacy concerns (β = -0.26, p = 0.000) was negative and statistically significant, and the effects of fear (β = 0.45, p = 0.000) were positive and statistically significant. These results are almost identical to the effects in the TRA model. However, whereas fear also had a positive and statistically significant direct effect on intent (β = 0.38, p = 0.000), privacy concerns did not (β = 0.01, p = 0.837). Privacy concerns effects on intent were completely mediated by attitudes. Experience with burglary also had an effect on intent (β = 0.11, p = 0.036), as was the case with the TRA model. The model explained 45% of the variance in intent to purchase an IoT security device and 27% of the variance in attitude toward purchasing (adjusted R^2^).

## Discussion

Using the TRA framework, we demonstrated that the intent to purchase IoT home security devices was about equally influenced by attitude toward purchasing and by the views of important others. Those important others included friends, family, neighbors and co-workers. If the respondent believed these people thought purchasing such devices was important, then the respondent had a stronger intent to purchase them. Attitude toward purchasing was based on beliefs about fear, related to home burglary, and beliefs about threats to privacy associated with security devices. Beliefs about privacy negatively affected attitude, but not as much as fear positively affected it. Despite concerns about an IoT home security device being associated with the loss of privacy, respondents were still driven by fear of home burglary to intend to purchase these devices. Concerns about privacy risks are not strong enough to undo the perceived benefits of IoT home security devices. To many consumers, the exchange of private information for security is worth the risk. In addition, experience with home burglary, whether of their own home or of others’, increased respondents’ purchasing intent.

Although the results supported all of our hypotheses and may seem obvious, they point to a more nuanced situation than might otherwise be expected. While fear was a major driver of attitude, and attitude drove intent, beliefs about the expectations of important others was just as important as attitude. This may be because the focus was on intent to purchase a very specific type of home security device–IoT devices–rather than home security devices generally. The views of important others may not be as important for devices that do not sit on one’s home network but rather are installed and monitored by third parties. While we found that privacy concerns were also important to attitude, their importance was no doubt enhanced by the public perception of IoT devices as “leaky” sources of private data. Dramatic examples of voyeur access to children via IoT devices, such as the Mississippi Santa Claus case mentioned earlier, may also have played a role in enhancing the primacy of privacy concerns. We did not hypothesize about the potential role of control variables, and only experience with burglary was statistically significant. This finding showed that intent to purchase was enhanced by direct or indirect experience with home burglary.

Our mediation analysis showed that privacy concerns were completely mediated by attitudes towards purchasing IoT home security devices. Although we did not have specific expectations about this relationship, our findings are intuitive. As we have discussed, IoT home security devices are perceived by the public as being sources of leaked personal data or as facilitators of evasions of privacy. If a consumer believed an IoT security device was not secure, their attitudes towards purchase would be weakened, and the consumer’s intent to purchase the devices would be lessened. Fear, on the other hand, proved to be a more general belief, mediated in part by attitudes, but also exerting a strong direct influence on intent to purchase. Fear could motivate a consumer to purchase an IoT security device, even if their attitudes toward purchasing such devices were not particularly strong. Privacy concerns, then, are dependent on the security technology in particular, while fear is less so.

From a theoretical perspective, TRA was shown once again to be a reliable, straightforward way to think about and study intent to engage in a particular behavior, this one being intent to purchase IoT home security devices. Although our central focus was on comparing the respective roles of fear and privacy concerns in affecting attitude, through relying on TRA, we also uncovered the importance of the perceived attitudes of others in formulating intent. By violating TRA’s prescriptions about the central role played by attitudes, and conducting a mediation analysis, we were able to learn more about the relationships between beliefs and intent. Future research that relies on TRA might consider including mediation analyses of some sort.

From a practical perspective, it seems marketers of IoT home security devices are wise to focus on fear for increasing sales. They are also wise to downplay or not mention at all any IoT capabilities that might be associated with compromised privacy. Our findings indicate marketers may want to enhance fear by playing on consumer experience with home burglaries. They may also want to invoke how important others would feel about purchasing IoT home security devices. A winning spiel might sound like “Home burglaries are more common than you think–they happen often and even in your neighborhood. Protect your home and family with XYZ IoT home security devices. Your mom wants you to keep her grandkids safe.”

As is the case with any study, ours had limitations. The subject pool was limited to American consumers. The sample size satisfied the requirement of five observations per model parameter, but a larger sample size would permit the exploration of other beliefs and their effects on attitude. Using the Theory of Planned Behavior rather than the Theory of Reasoned Action would have necessitated collecting data on perceived behavioral control. However, the target behavior was purchasing IoT home security devices, which is a fairly straightforward behavior, both online and in person, so perceived behavioral control would probably have not played a major role. There may also be differences in attitudes toward purchasing security devices that require third-party monitoring, as opposed to the types of devices we were studying. Each of these limitations could be explored in future research, with a non-American sample, a larger sample, a different theoretical framework, and/or a different device to be purchased.

## Conclusion

The global market for the smart home industry is projected to be worth over $150 billion USD by 2023, and a big component of that market is security devices, such as the Ring doorbell. The global market for smart home security devices alone is expected to exceed $5 billion USD by 2025. Fear is often used as a way to sell such devices, and we found that fear has a strong impact on consumer attitudes toward purchasing smart home security devices. Not surprisingly, we also found that security concerns about these devices also affected attitudes towards purchasing them. Attitude greatly influenced intent to buy, but what important others thought, and past experience with burglary, also affected intent. Attitude, subjective norms, and experience together explained 38% of the variance in intent. Researchers and marketers should take all three factors into account when exploring the major drivers behind purchasing IoT home security devices.

## Supporting information

S1 Appendix Survey instrument. (DOCX)
