# Supplementary material for: Intent to purchase IoT home security devices: Fear vs privacy
Source: PLoS One. 2021 Sep 21;16(9):e0257601. doi: 10.1371/journal.pone.0257601 (PMC8454981; doi:10.1371/journal.pone.0257601)
Supplement: S1 Appendix — (DOCX) [file pone.0257601.s001.docx]

**Internet-of-Things Home Security Survey Instrument**

**Modified and created by J.F. George, R. Chen & L.Y. Yuan, 2017**

**Attitude (George, 2004)**

Having an Internet-of-Things device is a:

Bad idea 1 2 3 4 5 6 7 Good idea

Having an Internet-of-Things device is a:

Foolish idea 1 2 3 4 5 6 7 Wise idea

Having an Internet-of-Things device is an idea I:

Dislike 1 2 3 4 5 6 7 Like

Having an Internet-of-Things device would be:

Unpleasant 1 2 3 4 5 6 7 Pleasant

Unless indicated otherwise, all items used this scale:

Strongly Disagree Agree Strongly

Disagree Disagree Somewhat Neutral Somewhat Agree Agree

1 2 3 4 5 6 7

**Normative structure (George, 2004)**

My neighbors would think that I should have an Internet-of-Things device.

My co-workers would think that I should have an Internet-of-Things device.

My family would think that I should have an Internet-of-Things device

**Intent (developed by the authors)**

I intend to buy an Internet-of-Things device before long.

I plan to add an Internet-of-Things device to my home network very soon.

Within the year, I expect to install an Internet-of-Things device.

I will obtain an Internet-of-Things device very soon.

**Fear (Milne et al 2000)**

I am worried about the prospect of my home being burglarized.

I am frightened about the prospect of my home being burglarized.

I am anxious about the prospect of my home being burglarized.

I am scared about the prospect of my home being burglarized.

**Privacy Concerns (Xu et al 2011)**

My family data could be inappropriately used by the provider of an Internet-of-Things device.

There would be high potential for privacy loss associated with allowing the provider of an Internet-of-Things device to access the recorded data of my family.

In general, it would be risky to allow the provider of an Internet-of-Things device to access the collected data of my family.

Providing the vendor of an Internet-of-Things device with the collection of my family data would involve many unexpected problems.

**History**

Has your home or that of someone you know been burglarized:

- In the past year

- In the past two years

- In the past three to five years

- More than five years ago

- My home, and the homes of people I know, has never been burglarized.

**Marker variable**

I prefer blue to other colors.

I like blue color.

I like blue clothes.

**References:**

George, J. F. The theory of planned behavior and internet purchasing. *Internet Research* 2004; 14(3) :198-212.

Milne, S., Sheeran, P., and Orbell, S. Prediction and intervention in health‐related behavior: a meta‐analytic review of protection motivation theory. *Journal of Applied Social Psychology* 2000; 30(1): 106-143.

Xu, H., Dinev, T., Smith, J., and Hart, P. Information privacy concerns: linking individual perceptions with institutional privacy assurances. *Journal of the Association for Information Systems* 2011; 12(12): 798.
